# International higher education scholarships: *a *pathway for Palestinians’ academic recovery

**DOI:** 10.1007/s10734-024-01271-5

**Published:** 2024-07-19

**Authors:** Anas N. Almassri

**Affiliations:** School of Education, Durham University, Durham, UK

**Keywords:** Educational development, Higher education, Graduate study, International education, Scholarship programs, Palestine

## Abstract

Scholarships offer *one* significant pathway for higher education recovery and development in Global South contexts. Although some research exists to illustrate this significance, the case of Palestine remains virtually unresearched. This article is a first contribution to bridging this gap. It draws on qualitative data collected through interviews with and pre-existing documents from 32 Palestinian scholarship alumni and alumnae. Four experiential themes emerged through critical realist thematic analysis of this data. Two of these themes are reported in this article. First, the participants reflected a range of negative and positive motivations for pursuing their funded graduate education abroad: escaping limited opportunities in Palestine, actualizing potential, and serving Palestine. Second, they described experiencing a mix of exciting and challenging (re)adaptations while appreciating new approaches to the content and practice of their academic learning. Together with the identified participants’ demographic and academic backgrounds, these thematic findings extend global empirical evidence of the contribution of international scholarships to higher education access, recovery, and development. They also avail a useful and timely frame of reference to inform future research and practice of higher education scholarships for Palestinians.

## Introduction

At the time of writing this article, higher education in Gaza is being destroyed (Jack, 2024). In a cycle intensified since 2008, “[u]niversity staff and students have been killed while campus infrastructure has been attacked, rebuilt, and destroyed again” (Milton et al., 2023, p. 1024). If we zoom out from the moment and region, we remain caught in a historical and national context in which Palestinian higher education seems to be forever hostage to the Israeli–Palestinian conflict. Its institutions were built under occupation and have since been deeply affected by the violence, politics, and ethos of the conflict (see Moughrabi, 2004; Newby, 2009; PCRR, 2021). If we zoom out even further, we know this political lethargy about the human enterprise of higher education is not unique to Palestine but has recently come to characterize political violence in the Middle East and beyond (Bennouna et al., 2018; Milton et al., 2023). At chronic risk in this context is the compounded issue of *safe access* to *quality* higher education, i.e., Palestinians’ ability to access good opportunities of higher education at home without threats of injury or destruction to their lives or academic institutions (see GCPEA, 2022; Snounu et al., 2019).

My argument here is that *one* pathway for mitigating that risk is international scholarships. The article provides a first, timely study of Palestinian students’ motivations for and experiences of academic capacity-building through funded graduate education abroad. While conceding that scholarships are but one such risk mitigation pathway, the article demonstrates that they can facilitate inclusive and successful investment in (re)building cadres for Palestinian higher education. Crucially, neither this article nor its parent project, a doctoral study, had been planned as a response to the cycle of violence that commenced on October 7th, 2023. However, the discussion is timely in providing an empirical background to guide responses to Palestinians’ increased need for and interest in higher education abroad, including amidst calls to expand scholarship opportunities for them (see Kasraoui, 2024; Neve Gordon, 2024). To this end, the article reports 32 Palestinian scholarship recipients’ background characteristics and their various motivations for and academic experiences through master’s programs abroad.

### Setting the scene

Higher education systems around the Global South have benefited from international scholarships. Kazakhstan’s government scholarship alumni reported improving assessment systems, fostering academic integrity and a research culture, and training and mentoring colleagues at their universities as well as promoting education and employment opportunities in their social circles (Jonbekova, 2023; Jonbekova et al., 2023). Turkish alumni of the US Fulbright scholarship program reported adopting pedagogical changes, contributing to curriculum reform and development, and launching new programs, including through collaboration with foreign departments (Demir et al., 2000). Similarly, Saudi academics who were recipients of the King Abdullah Scholarships reported enhancement in their personal effectiveness, English language competency, career progression, and teaching and research skills (Pikos-Sallie, 2018). In Ghana and Nigeria, alums of the Ford Foundation’s International Fellowships Program reported clear(er) plans for using their education abroad to disseminate knowledge that supports positive change and national development and, like Kazakhstani alums, more broadly serving as public advocates of inclusive educational opportunities (Campbell et al., 2021). Extant empirical evidence, though scarce and temporally and thematically limited, suggests similar benefits of scholarships to higher education in Arabic-speaking countries. Historically, at a time of few universities regionally and unaffordable ones abroad, scholarships by the Mandate governments of Iraq, Palestine, and Transjordan facilitated “intellectual pilgrimage” to the American University of Beirut in Lebanon, graduates of which went on to lay the foundations of higher education in and across countries of the region (Kalisman, 2015). Though for school students, Kuwait’s Arab Scholarships in the 1950s helped qualify prospective leaders from across the region for higher education and the labor of achieving independence (Al-Rashoud, 2019). Today, Saudi Arabia offers its students the world’s largest program of outbound scholarships as the Kingdom, like the neighboring United Arab Emirates, seeks advancement and innovation in its own higher education, economy, and political standing (Hilal, 2013).

As these cases suggest, local and foreign, and public and private entities worldwide invest significantly in international scholarships (see Dassin et al., 2018). They often do so with the objective of building capacities for national or foreign higher education in the Global South. This objective overlaps with what Campbell and Neff (2020) systematically identify as larger purposes of international scholarships, e.g., human capital development, diplomatic influence, and/or development aid (also see Campbell & Mawer, 2019). It also seems to overlap with a spectrum of other (un)intended or less clear objectives for scholarships, to extend colonial/hegemonic ways of knowing (Chiappa & Finardi, 2021; Thayer, 1914; Ziegler, 2008), to maintain authoritarian stability (Del Sordi, 2018), to manage domestic political and economic affairs (Pavan, 2020), and to expand global solidarity (Campbell & Neff, 2020). Despite the recent growth of analyses of and critical arguments about scholarships impact (e.g., Campbell & Mawer, 2019; Saling, 2023), the lack of standardization in levels of impact assessment, enormous variety of evaluation methods, and confusing mix of theories warrant serious caution before pronouncing any judgment on *a* prevalent effect or impact trajectory of scholarships (Mawer, 2018; for illustration, see Novotný et al., 2021). I find the case of scholarships to Palestinians particularly interesting to locate amidst this body of evidence of scholarships’ impact but also amidst arguments of scholarships’ value in such volatile contexts as that of Palestine. Below, I begin to establish some context for scholarships to Palestinians, acknowledging that this context itself requires a dedicated research project and a discussion beyond the scope of this manuscript.

Historically, Palestinians pursued their higher education abroad, due to forced displacement amidst cycles of the Israeli–Arab conflict and/or to lack of local higher education until the 1970s (Abu-Lughod, 1973). In this historical context, only Palestinians of economic and/or political privilege accessed higher education abroad (Al-Hout, 1979; see Bruhn, 2006, for more on the history of Palestinian higher education). While no recent data exists on Palestinians’ funded academic sojourns, Elhour (2022) suggests that in the past few years, a new wave of Palestinian academic mobility could be observed, one she attributes in part to increased availability of scholarships, whether offered specifically for Palestinians or inclusively of them. Indeed, Palestinians today are eligible to compete for scholarships funded by the governments of Belgium, France, Germany, Hungary, Japan, Turkey, the Netherlands, the UK, the US and a few Arab countries like Algeria and Morocco. In the UK, they are also offered annual graduate scholarships like the Hani Qaddumi Scholarship Fund^1^ and the Saïd Foundation scholarship.^2^ Also, initiatives to advise Palestinians on applying for American/Western undergraduate institutions continue to be led by AMIDEAST,^3^ an American INGO active in the field of international education, as well as by Palestinian NGOs like the Welfare Association.^4^ While neither these scholarship programs/initiatives nor their impact has been studied, a rapid review of their announced objectives confirms the centrality of capacity-building for higher education amidst a web of other objectives, from intercultural dialogue and diplomatic influence, e.g., foreign government-funded scholarships like Chevening and the Fulbright, to political solidarity and academic humanitarianism, e.g., DPET,^5^ STEPS,^6^ and OBGS.^7^ For example, HESPAL,^8^ Higher Education Scholarships for Palestinians, is a British Council-administered, multilaterally-funded scheme for graduate and doctoral study in the UK aimed at creating “the next generation of senior academics who can maintain international quality standards at Palestinian universities and develop renewed and sustainable links between Palestinian and UK universities.”

Amidst the global scene of growing scholarship programs and research, we have in Palestine a local context of historically strong interest in scholarships, of contemporary increase in scholarship opportunities, and now of urgent need for bridging the chronic research deficit on the value of these opportunities for the recovery and development of local higher education, among other sectors. This article, the first of a series of three, begins to locate the case of scholarships to Palestinians amidst the global context of higher education scholarships. The findings reported here illustrate how funded foreign education abroad may well contribute to higher education recovery and development in Palestine. They also add to extant research that highlights the positive contribution of scholarships to higher education development in various contexts, including those of protracted conflict and under-resourced academic institutions. The article is dedicated to describing 32 Palestinian graduate students’ motivations and academic experiences through their various scholarships. Throughout the article, theoretical reference and academic jargon are deliberately kept to a minimum for two objectives: to make the article accessible to a broad audience of academics, practitioners, and others interested in scholarships for Palestinians, and to fulfill the requirement in critical realist philosophy for distinguishing perceptions of reality from inferences about it and explanations of it (Bhaskar, 2016). I discuss below the methodological approach to achieving both objectives.

## Methods

### Sampling and data collection

I present here the research methodology segment from the parent project that is relevant to this article. To begin with, purposeful sampling was followed in recruiting participants who met all these criteria: Palestinian citizenship and residency in Gaza or the West Bank, undergraduate education in Palestine, receipt of a master’s scholarship, and undertaking with it in-person study at a higher education institution abroad, and graduation between 2016 and 2022, i.e., 1–6 years before the time of their research participation. Thirty-two participants participated in this research through semi-structured, in-depth interviews. They were prompted to share their perceived experiences and outcomes of education abroad and their post-completion engagements, as well as their demographic, academic, and career backgrounds. Interviews lasted on average for 65 min and were conducted online via Teams in Arabic and/or English. Sixteen completed a participant background form where they shared more contextual data about their backgrounds; eleven further shared one or more relevant pre-existing documents—mostly a copy of their CV/resume and completed scholarship application form. Data was collected from January through March 2023.

### Data analysis

Interview recordings were transcribed via Teams, reviewed and edited manually for accuracy, and where appropriate, translated to English. All pre-existing documents provided were confirmed for their scientific quality against Scott’s four criteria of authenticity, credibility, representativeness, and meaning (Scott, 1990, cited in Mogalakwe, 2009, pp. 51–56). Next, all data items were uploaded to NVivo, where they were further annotated with initial reflections.

By applying Wiltshire and Ronkainen’s (2021) method of critical realist thematic analysis, I committed to being data-driven in exploring empirical patterns in the participants’ reported scholarship experiences. First, I carried out a dual step of data-driven, descriptive coding of interview transcripts and pre-existing documents. I labeled certain parts of text in these sources based on whether they described closely similar experiences, and I checked whether resulting data-driven codes were available in other participants’ data items. Using NVivo allowed me to operate this process efficiently, with the guiding principle of descriptive validity (Wiltshire & Ronkainen, 2021): If a new excerpt offered descriptive insight *and* this descriptive insight is represented in an existing data-driven code, I added the excerpt to that code. Second, I built descriptive patterns through integrating data-driven, descriptive codes of significant empirical relevance to each other, in course checking resulting patterns for prevalence and meaning-significance (Braun & Clarke, 2022). Finally, descriptive patterns, along with reactions and reflections recorded on NVivo while coding, were developed into experiential themes. Nascent experiential themes were first developed by consolidating descriptive patterns of significant empirical relevance to one another. I then revised these based on whether and how well they demonstrated an empirically coherent class of thematic findings and checked them for empirical adequacy, descriptive validity, and interpretive validity (Wiltshire & Ronkainen, 2021). To illustrate, some participants indicated in their interviews and in their CVs taking modules that spanned different disciplines; these interview and documentary indications were all added to a descriptive pattern of “appreciating interdisciplinary learning,” which was subsequently developed as part of the theme on the participants’ academic journey (presented later in this article). Here, it is worth noting that because only a third (34%) of participants shared pre-existing documents, documentary data served a primarily supplementary purpose, i.e., confirming, deepening, and/or broadening the participants’ input through interviews.

Two of the four experiential themes that emerged from this process are presented in this article. In critical realist research, “experiential” themes are the first layer of themes; they offer a descriptive summary of the layer of reality that is subjective and which can be directly accessed by, inter alia, reported perception of a phenomenon (Fryer, 2022; Wiltshire & Ronkainen, 2021). That is, experiential themes represent prevalent and significant segments of the participants’ reported scholarship experiences. Because of their descriptive nature, these themes are fit for the purpose here of exploring the participants’ scholarship experiences vis-a-vis two research questions: What are some of the profile characteristics of Palestinian recipients of international scholarships (RQ1)? How do Palestinian scholarship recipients perceive their motivations for and experiences of undertaking funded graduate education abroad (RQ2)? Although abstract terms had to be used in these themes, such terms should be read with their generic rather than any theoretical reference.

### Notes on methodological approach

The preceding data analysis strategy enabled me to produce a “descriptive summarization” (Da Wan et al., 2022) of the 32 Palestinian scholarship recipients’ experiences. This summarization contributes a first frame of empirical reference for the topic of scholarships for Palestinians, one that informs future research and is accessible to scholarship program administrators. Before presenting the findings, three points are worth noting here. First, I pursued description in this article independently of any attempts at theorization or explanation, which form later methodological stages of critical realist research (Wiltshire & Ronkainen, 2021). It will be in those two later stages where I deepen my data analysis and, among other tasks, infer what applications of individual agency (in face of what oppressive and conducive structures) the participants’ data demonstrate they followed through planning and undertaking their funded graduate education abroad. Second, this delineation of the descriptive from the inferential is not only a matter of fulfilling philosophical-methodological assumptions of critical realism (Bhaskar, 2016). It also helps fulfill the essential, albeit often underestimated, role of description in social science research (Gerring, 2012), a role most crucial to building a baseline understanding of a topic as unresearched as scholarships for Palestinians. As Holmes et al., (2024, p. 51) contend: “Descriptive research—work aimed at answering “who,” “what,” “when,” “where,” and “how” questions—is vital at every stage of social scientific inquiry.” These are the questions to which a response is commenced in this article—and extended in the remaining two articles of this series (Almassri, 2024a, 2024b). Finally, the delivery of this methodological approach benefited greatly from my insider status as a Palestinian scholarship recipient myself, formerly a practitioner of international education in Gaza and currently a doctoral candidate in education. This status helped me particularly with accessing the participants, securing their interest in (often elaborate) contribution to the research, and following a context-driven approach to identifying the meaning-significance of some of their input (see Almassri, 2023).

## Participants’ profiles

This section presents key aspects of the research participants’ profiles. In terms of demographics, 81% of the participants are from Gaza and 19% from the West Bank. They undertook their funded graduate education abroad between the ages of 22 and 31, with women making up 60% of them and men 40%. Of the 16 participants who further completed the Background Form, six self-reported coming from an upper-middle-class family, two from a lower-middle-class family, and five from a working-class family. Nine reported having attended a primary school (grades 1 through 9) run by UNRWA, the UN agency for Palestinian refugees. Thirteen reported having attended a public high school (grades 10 through 12), compared to three who attended a private high school.

**Fig. 1:**
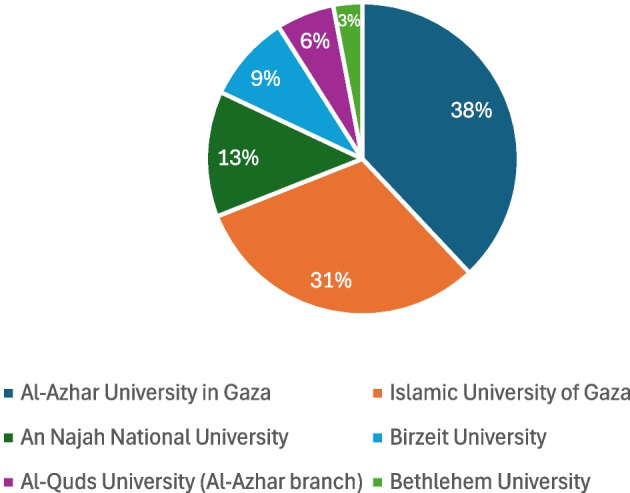
Participants’ institutions of undergraduate study

Academically, the participants represented a diversity of trajectories. Their undergraduate programs spanned languages (47%), mostly English but also French, medicine and related health programs (25%), engineering (9%), business (6%), law (6%), journalism (3%), and mathematics (3%). The prevalence of academic backgrounds in languages and in health-related fields potentially reflects the predominance of these fields as common choices for highest-achieving Palestinian school leavers. The participants pursued their undergraduate programs in these fields at six different Palestinian universities (see Fig. 1). Given the prohibited mobility between Gaza and the West Bank, I take the prevalence of graduates from Al-Azhar University in Gaza and the Islamic University of Gaza to reflect the greater representation in the sample of Palestinians from Gaza than those from the West Bank, as well as the status of both as Gaza’s leading universities, i.e., the ones to which highest-achieving high school graduates tend to go. Twenty-eight of these participants, for whom data was available, waited on average for 2.4 years between completing their undergraduate degree and starting graduate study.

In their graduate study, 18 participants (56%) pursued specializations in their undergraduate fields, and 14 (44%) shifted their fields of study. Examples of specialization include participants with an undergraduate business degree undertaking graduate programs in marketing or international business. Examples of shifting fields of study include participants who shifted from dentistry and pharmacy in their undergraduate study to anthropology and development in their graduate study and others who shifted from English to social work, international relations, and global development. Over two-thirds of the participants pursued graduate programs that involved more than one social science discipline; the single greatest prevalence (19%) was of programs in or heavily drawing on international relations, e.g., International Relations, Human Rights and Conflict, International Law (Human Rights), and Global Health (Conflict and Security).

The participants’ study destinations spanned Australia (3%), England (66%), France (9%), Jordan (6%), Qatar (6%), Scotland (9%), Switzerland (3%), and the US (6%) (note the total is greater than 100% because three participants held two different scholarships and studied in two different countries). The greater representation of participants who studied in the UK, 87% of whom attended Russell Group universities, reflects their more positive response to the research participation invitation (a 92% positive response rate), my greater access to them, and potentially one or more of the following factors: the prevalence and comparatively greater awareness in Palestine of scholarships to study in the UK, preference for the relatively shorter duration of master’s programs there, and the reputability of British higher education (Elhour, 2022, pp. 139–146; also see UCU, 2017).

**Fig. 2:**
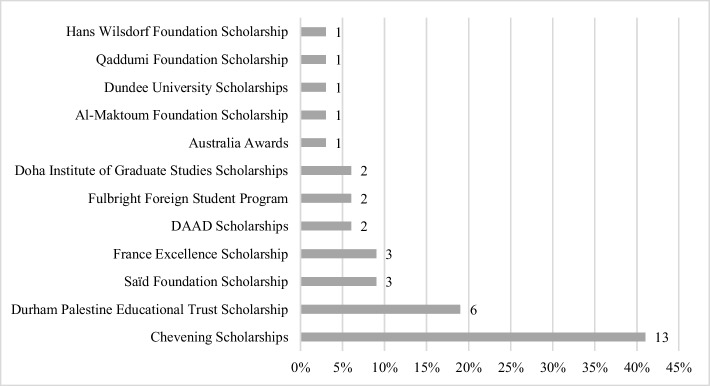
Scholarships awarded to participants

The participants’ scholarship programs spanned different categories of primary funding from foreign governments (65%), private foundations and charities (37%), and universities’ institutional aid (9%) (see Fig. 2). Seven participants (22%) reported securing more than one scholarship simultaneously. One of them combined their two full scholarships into a joint one. This, together with the three aforementioned participants awarded different scholarships, raises the total number of scholarships awarded to the participants from 32 to 36 as presented in Fig. 2.

In sum, these profile findings of the participants’ trajectory of academic excellence, their pursuit of specialized or new study, and their competitive access to full funding mean that they were strongly positioned to draw significant gains of their funded graduate education abroad. The first experiential theme below further describes the participants’ motivation to pursue graduate education abroad.

## Motivation powered by ambition and pragmatism

In their interviews and pre-existing documents, the participants demonstrated various motivations for choosing to study abroad and for choosing their scholarships, study programs, and countries of study. Descriptive analysis of their data shows these (sometimes gendered) motivations range from negative ones, e.g., escaping limited or poor opportunities in Palestine, to positive ones, e.g., academic and career advancement and service to Palestine. In pursuing these intentions, the participants reflected a pivotal role of both scholarships and past engagements in shaping their study program and destination choices.

### Seeking possibility and advancement

Several participants articulated that they were driven to study abroad to actualize more of their perceived potential for academic and career progress and for serving Palestine. A participant who studied tourism and sustainable development in France and had worked in Palestine’s precarious tourism sector (Isaac et al., 2019) said:

I had been working with *Masar Ibrahim*, a community tourism project that offers domestic and foreign tourists a walking path from the north to the south of the West Bank, in course learning about local communities and their heritage and traditions. My plan [of studying abroad] was to come back and continue working in this sector and build something related to sustainable tourism, especially environmental tourism. (translated)

Two other participants said their pursuit of a master’s followed their longer-term plans to become academics in their respective fields, mathematics and literature. Both said their passion for academic work followed their success as undergraduate students, an early time when both took language tests and other preparation steps to apply for scholarships to advance their education abroad.

Women participants offered often a gendered expression of similar aims of career and academic advancement. In explaining her graduate program choice, international relations, one participant said it had been a decision long in the making:

My passion is politics, not journalism. I studied journalism [in my undergraduate degree], but my eyes were on politics. I did so because it was clear to me that it was difficult for a fresh woman graduate to work in politics or get published writing political columns. (translated)

Another participant reflected more clearly on her gendered, critical motivation behind also choosing to study international relations:

When I was thinking about the master’s, I just thought how crucial this program would be to my community… I just thought we just need this—and also we, especially like females, we don’t see females playing a role in Palestinian international relations. In fact, even the males who are doing this, I don’t, I personally don’t believe they’re doing the job really well.

Beside this ambition for advancement, a quarter of the participants said leaving (more often Gaza than) Palestine, whether temporarily or permanently, was *a* or *the* key force of their motivation to pursue graduate education abroad, primarily to build their capacities in their fields of interest. One participant, a medical doctor with specialized interests in fulfilling which Palestine offers no appropriate training or work opportunities, said:

I was applying for a master’s scholarship, *any* master’s scholarship, because I was just at some point escaping from Gaza. And at some point that was the only motivation that I had personally, that I need to get out of this place, because I cannot actualize my potential in this place. And I knew that I have some potential that needs to be actualized somewhere else. …

Similarly, another participant from Gaza had studied English and French in Palestine but felt frustrated at what she described was her education’s lack of meaningful experiences and work-related skills. She said her frustration grew when she started developing interest in humanitarian work but found neither relevance to this field of her language and literature studies nor academic training in Gaza to prepare her for doing professional humanitarian work. Therefore, she said, she “spent five years just focused on trying to work or volunteer at humanitarian organizations, e.g., the Red Crescent, while also being focused on finding a scholarship to study this field abroad”. Repeatedly using the collective voice, a third participant, from the West Bank, said:

We left because home right now offers no hope, and we’ve remained abroad because we can’t afford to go back and be unemployed, let alone survive violence of the occupation and of settlers. We’re abroad, but we know we’re building the capacities we know we’ll bring back home once conditions there improve. (translated)

### A pursuit dually shaped

In recounting how they went about pursuing these plans, nearly all the participants reflected that availability and conditions of funding as well as prior academic, career, and/or civic engagements (greatly) influenced their scholarship, study program, and country choices. For example, in explaining her application to the US Fulbright Foreign Student program, one participant said:

I’m going to be honest with you. I didn’t know what the Fulbright was. All I knew it was a scholarship, and it would pay my tuition if I went abroad. I had no idea it was this competitive, had no idea it was this prestigious. I just wanted to try again because I applied for the first scholarship in my senior year in undergrad, and I actually got this scholarship to Canada, but I couldn’t leave Gaza because of the political situation in Egypt in 2013.

Similarly, many participants said they applied to more than one scholarship, whether in a single year or across 2–4 years, to ensure they had higher chances of success and to start their graduate study at their intended time. Following this line of reasoning, the participants followed in their choices the significant influence of conditions associated with available scholarship. For example, for a third of them, the scholarships they ultimately secured could be held at only specific institution(s), e.g., DPET for Durham University in the UK.

This reported influence of scholarship availability on the participants’ choices follows (their recognition of) the indispensability of financial support for their ability to undertake graduate education abroad. One by one, the participants reported in interviews that they would not have been able to afford the travel costs, living expenses, or tuition fees of their graduate education abroad without the scholarship funding. One participant, who studied in France, was particularly elaborate (and critical) in his account of how “unthinkable” these costs are for most Palestinians:

Let’s be frank and realistic. Who from Palestine can afford self-funding their studies abroad? Only the wealthiest; the most elite; children of Palestinian Authority top executives, of ambassadors, of businessmen. The class to which I belong—and to which most Palestinians do—has to work all their lives to manage their living costs. I mean, we had to borrow our way through undergraduate education at local universities [where the annual cost of attendance may range from 800USD to 5,000USD]. It’s just not even thinkable for me to have afforded my education abroad. (translated)

Documentary data further illustrated the indispensability of financial support for undertaking graduate education abroad. In her scholarship application, a participant who studied in the UK responded to the following prompt on financial need as below:

Prompt: How did you finance your first-degree course? (e.g. scholarships, employment, family)

Answer: My family paid for my first and second semester at Birzeit University. Since my second semester at Birzeit university until I graduated, I have always been on the honour list and this means that in the given semester I was able to obtain an average above 85% with no individual course average below 80%. This, in Birzeit university gave me an automatic tuition fee exemption for the following semester.

To further illustrate in contextual terms, even before the current devastation in Gaza, Palestine’s GDP per capita stood at 788 USD—with Gaza’s at 315 USD (PCBS, 2023). In comparison, the research participants could have faced dues totaling upward of 75,000 USD to undertake their graduate study in the US, 45,000 USD in the UK, 30,000 USD in Qatar, 21,000 USD in France, and 11,000 USD in Jordan.

Although potentially limited by this absolute reliance on available scholarships, the participants seem to have still drawn significantly on their past experiences in trying to shape their country and study choices. Most said they arrived at the application process following records of academic distinction, internship or career success, community volunteering, and/or other significant engagements and reflections beyond academics and work. In making their scholarship applications and deciding on their study programs, many participants drew directly on these records of past experiences. In their scholarship application, one participant wrote:

Working as a medical coordinator at [name of organization redacted] has given me a front row seat to examine and scrutinize the devastating defects within the health system. I lack the proper knowledge, tools and research methods to come through with my ambitious vision for a better health system. I realize my need for specialized studies into Global Health Science, Epidemiology, trends and updates in Communicable & Non-communicable Diseases, Clinical Trials and Meta-analysis, which are offered by the programmes I chose, MSc Global Health at the University of Glasgow, MSc Public Health at Oxford Brooks University and MSc Public Health at the University of Southampton.

Another participant explained the relevance of her academic achievements, career experiences, and gendered national identity to her intended study program. In her scholarship application, she clarified: “I believe that my background is the driving force behind my decision to apply for the LLM in International Humanitarian Law and Human Rights.” In another essay of her scholarship application, she referred to herself in the following order: “Arab Palestinian,” “woman lawyer,” “proficient in three [world] languages.” Clear links could be observed between this self-conception and her following vision for the future of her field:

Massive human rights violations are committed in addition to grave violations of IHL, in Yemen, Syria, Iraq, or Libya … In my opinion, there is a huge reliance on international experts in these areas [International Humanitarian Law and Human Rights], while we also need more indigenous human rights lawyers and experts who can not only use IHL framework effectively, but also translate these frameworks not only linguistically, but also culturally and politically.

It is worth noting that this sector is highly dominated by men. … I want to be able to break any social limitations to reaching my career goals as a humanitarian expert… and eventually, the social pattern that this job is reserved for men will be broken.

Similar patterns of motivation were noted in the interview transcripts of most participants. In the case of two participants, their undergraduate training was in the medical field and most of their wider engagements were in community service and charity work. Both said they pursued graduate programs in the social sciences to gain knowledge and skills better aligned with their past and intended civic and professional goals. For a third participant, her pursuit of an International Business specialization followed her undergraduate training in business, internships with various international businesses, and work in the export industry.

This first theme has presented the participants’ reported ambitions and pragmatism in planning and pursuing their funded graduate education abroad. As the following, second theme demonstrates, these motives were mostly fulfilled through the participants’ academic experiences abroad.

## A journey of academic (re)adaptation and fulfillment

This second theme reports the participants’ experiences of exciting and challenging (re)adaptations while appreciating new approaches to the content and practice of their academic learning. This theme shows the participants struggled with but often appreciated and overcame the demands of graduate-level study in a foreign academic environment.

### A challenge met: research reading and writing

Several participants began their accounts by describing initial and/or constant struggles in dealing with the quantity and quality of (research) reading and writing in their study programs. One participant, a graduate of English and Education, said of the following of this struggle:

It was, it was torturing for me because I never really in my life made research. I never read a scholarly article in my life. I graduated from Birzeit but without really reading any academic paper in my life. So, it was hard, very hard to read, to write, to be critical, to keep up with what they’re talking about. And another thing is that like how to do research, how to write, how to be critical.

For another participant, the challenge was harder because his program in France was delivered in academic French, which he felt was different from the French language he learned in his undergraduate study in Palestine.

Specific research writing tasks were particularly challenging for several participants, e.g., organizing one’s research writing, completing (critical) literature reviews, and articulating meaningful interpretations. One participant, who studied in the UK, experienced a mix of stress about writing such assignments and regret at not having been exposed to them in their undergraduate education in Palestine:

I remember while stuck writing one of my summative essays, I felt stressed about it, and I shared a Facebook blog on why our universities never introduced us to written assignments! I mean, all our assessments were of exams and tests, which didn’t help us learn how to write or do these kinds of written assessments. (translated)

Another participant, who studied in Qatar, reported the same challenges but commended her institution’s approach to supporting students through offering courses on the basics of academic research.

Despite these challenges, the participants reported usually feeling appreciative of the contribution they perceived of writing-intensive learning tasks to developing subject-specific skills like technical report writing and broader intellectual skills like critical and analytical thinking. A third of participants categorized the academic writing skills they gained during their graduate education abroad as among the most valuable parts of their experience. Even for the two participants whose “torturing” and “new language” experiences were presented above, they evidenced such appreciation by recounting good progress through their current doctoral study in France and the UK.

### Perceived learning: research-oriented, interdisciplinary, and global

In terms of substantive learning, many participants spoke mostly highly of their learning attainment in their subject areas, specifically focusing on the research-oriented, interdisciplinary, and global scope of their learning content. For some participants, the methodologies and theories introduced in classes and readings helped them develop more critical and systematic reflections. One participant, who studied in the UK, particularly appreciated their exposure to participatory action research as their prior community service and charity work followed no academic background in social science:

When I studied participatory action research, I was like, I have been doing this all my life, but I never thought it had a term! Even for the ethics of it, I just thought that my charity work was benevolent and thus “ethical” and so didn’t require much thinking about its ethics. Now, I can better organize my thoughts about my work, and I keep reading others’ works on participatory action research to see how to improve the ethics and success of my work. (translated)

Another participant’s study of international relations in the US exposed them to various schools of thought in the field. They were drawn to the realist school, an interest they further advanced following reactions of shock or condescension from American and European classmates:

I remember we were having this conversation about how everyone subscribes to a certain IR theory, and when it was my turn, I said I was a realist and everyone laughed, “how could you be a realist!” They just thought because I am from the Middle East, I should be subscribing to postcolonialism or one of those liberation theories. They found it almost shocking that a Middle Easterner believes in realism.

The participant said they dwelled on these comments before growing more affirmative in their subscription to realism: “The theory kept making sense to me, and every time I read more about it, like, I just don’t believe in the whole thing of human rights, the United Nations, which, you know, wash all the scandals that governments do.”

Also, some participants articulated that their interdisciplinary learning was of great value to their subject knowledge development. This was especially the case of participants pursuing specialization in their fields of undergraduate study. For one participant, a medical doctor by undergraduate training, his Global Health graduate program focused on conflict and security. He appreciated this interdisciplinarity because it helped him “better see global health from a security perspective and from the standpoint of international politics.” Similarly, three participants with undergraduate training as engineers who specialized further in their respective fields offered similar, highly positive accounts of considering social and/or political issues while building technical solutions. One of these engineers said:

My study [in Palestine] was purely technical. It was all of numbers, statistics, and technical drawings. The social aspect [of engineering] was absent. For example, in environmental engineering, we once worked on an assignment to extend sanitation works to more areas in Gaza. Our work involved technical analyses—determining water levels, ground height, pipeline length, and manhole location. But we never considered the social aspect of these analyses or the social impact of implementing our results. Unlike my program in the UK, programs at our universities never focus on this aspect. (translated)

Beside interdisciplinary learning, global learning was also a salient point of appreciation among the participants. In their interviews and pre-existing documents, many participants highlighted experiences of global learning in classrooms, in major learning projects, and beyond academic settings. For several participants, their readings involved case studies from “far” countries, and their class activities featured discussion of their (international) peers’ diverse perspectives on learning topics. Reflecting on her learning with/from international peers in a course on Data and Society, one participant said:

The content was about how data is used in China. China may sound to us [Palestinians] as an isolated, unheard-of place. In the course, I had the opportunity to read research but also to listen to opinions from many Chinese classmates. It was an interesting class because there were some differences between what claims we read in the assigned research, produced by Western researchers, and what descriptions my Chinese classmates gave of the use of data in their country. The gap between these research claims and my peers’ descriptions made me spend much time thinking before drawing a conclusion from a reading and while doing research. (translated)

This appreciation was also of the global scope of the participants’ completed learning projects. Some of the examples cited during interviews included group presentations and team projects where the participants said their work with global peers involved learning new communication skills and, again, diverse perspectives on their assigned topics. Documentary and interview data further evidence this appreciation. In their theses, two participants looked at the global context of Palestinians’ access to international exchanges and to foreign aid; a third participant focused on Germany and Turkey in researching the cultural memory of genocide, a fourth on Iran and Turkey in examining policies on gender-based violence, and a fifth on Jordan as their case study of water and sanitation services. All these participants reflected finding motivation to pursue these global learning projects in the international research resources available at their universities and the relevant regional expertise of their supervisors.

Additionally, the opportunity to meet people of other nationalities—most often for the first time—was also of reported value to participants’ global learning. With her graduate study focused on conflict and humanitarian work, one participant said she appreciated her interactions with Syrian and Yemeni students and communities in Qatar: “I heard from them firsthand stories of the crises in their countries, but I also learned a lot about the two countries, which we as Arabs assumed we knew but pretty much did not!”.

### Learning resilience and support

While navigating the aforementioned challenging and enjoyable learning experiences, the participants seem to have developed a new approach to their academic learning and their perspective on academia. Many described exerting “high” or “unprecedented” individual effort in progressing through their studies. A participant trained as a medical doctor said it took him “two months to realize what this new system required in terms of studying on my own,” following which he spent 12 days going to the library from 8 am to 10 pm to get his first writing assignment done. In their interview, the participant went on to explain that as he progressed through his master’s, he felt he got used to this amount of independent study. “I was assured of my progress when I saw my second-term grades,” he said. “Instead of 63 and 67 [in my first term], I now got 90 and 91, and I actually got distinction in my thesis.” Another participant, who studied in France, offered the illuminative example below of the time and (peer learning) effort he had to put in to overcome the language challenge ahead of written exams:

I used to gather notes from my peers, study them, predict what questions may be asked in the essay exam, draft a few essays based on readings and my own thinking, memorize them, and try to use them in answering the essay questions at the time of the exam. (translated)

For some participants, who were studying between 2020 and 2021, this amount of study seems to have become a significant challenge to their academic progress and/or well-being when it coincided with the social isolation caused by COVID-19. During England’s second national lockdown in late 2020, one participant said he struggled to maintain his energy and focus while studying alone in his student accommodation room. Another felt “highly frustrated and disappointed by the very low level of interactions [he] was able to do during the first four or even five months in the UK.” Others seem to have felt frustrated but quickly found ways to maintain their academic progress. A participant who completed her master’s with distinction in the UK in September 2020 offered the following account of her coping with COVID-19 impact:

COVID disrupted everything for me. I was making good progress; I had planned, and got support for, my international fieldwork for my thesis research. When COVID happened, I couldn’t even leave my dorm but for essential matters, let alone travel internationally. I had to re-plan my whole thesis research, which I did. It became a systematic review that I completed from my dorm. (translated)

Beside such resilience, being in supportive academic environments was also cited by many of them as enabling their quality progress. Two participants said they always found their professors available for and actively interested in further discussion of learning content. For two more participants, who studied in Qatar, they recounted enjoying their interactions with their professors. Both cited finding inspiration in these interactions, including when their professors—with academic and lived experience of regional affairs in the Middle East—challenged them to be more self-critical in their approach to their learning. With a consistent smile and a dynamic tone, one of these participants recounted the following powerful example of such interactions with one of his professors at the Doha Institute of Graduate Studies:

Professor [name redacted] said like, it’s one of the main questions in the field to define what the genre is, to label texts to a certain genre. I raised my hand and we kept debating why, why we need to label a text—I could write a short text and call it a poem or a short story. It’s up to me, the writer, not the reader…. Why should there be one specific form for the poem or for the short story and so on. Then, he started yelling, ‘ah, you Palestinians, because you don’t have a father, don’t have leadership, don’t have one single entity that you can turn to, you want to destroy the system; you don’t want institutions.’ He wasn’t insulting me, of course; he was analyzing. And he was right; we hate institutions; we don’t trust establishments, every time and everywhere we go, and it’s the same in our studies. We don’t like institutionalization.

Others in the participants’ environments were also supportive. One participant was generic in his appreciation: “It was my first-ever time to travel, so I assumed academics would be the hardest part of it, but I felt it was much easier than I had expected because people in the university were welcoming and supportive.” For four of the six participants on the Durham Palestine Educational Trust scholarship, they reported finding additional academic support in their conversations with one of the charity’s members, a retired Durham University professor.

## Concluding discussion

Findings reported in the previous section document some of the profile characteristics, motivations, and academic experiences of Palestinian students undertaking graduate education abroad on scholarships. This documentation directly contributes to addressing the knowledge gap of Palestinian scholarship recipients’ profile characteristics (RQ1). Profile findings suggest that Palestinians selected for international scholarships tend to be relatively young and recently graduated and to include men and women coming from diverse socioeconomic backgrounds and succeeding in various graduate disciplines and education systems. The findings demonstrate that Palestinian scholarship recipients, whose academic backgrounds were concentrated in STEM and language fields, go on to pursue more diverse areas of study, whether specialized or new to them but often with a global outlook. These findings extend limited available insights about the characteristics of Palestinian scholarship recipients that are found in Elhour’s (2022) doctoral thesis, Akbaşlı and Albanna’s (2019) conference paper, and marginally in relevant journal articles (e.g., Long, 2006).

The findings here also contribute to building knowledge of Palestinian students’ perceived motivations for and academic experiences of funded graduate education abroad (RQ2). The participants brought into their scholarship and education abroad planning a reflexive performance cross-cutting through individual ambitions, national circumstances, and international opportunities. Their ambitious self-actualization agenda, while often rooted in past local engagements, grew beyond local, and often gendered, academic and career possibilities. Still, the notion of—if not commitment to—serving Palestine seems to deeply characterize this agenda, even when it is reportedly put on hold until conditions allow for safe, sustainable, and productive return. This reflexive exercise between the personal, the national, and the possible is hardly surprising in the specific context of Palestinians’ academic sojourns abroad (see Kalisman, 2015; Long, 2006). Following their motivation, the participants found much value in their academic learning, including when and especially because it challenged them to scale up their intellectual practices and knowledge repertoire. The elaborate description here of Palestinian students’ academic gains and challenges deepens Elhour’s (2022) account of sociolinguistic gains and challenges reported by Palestinian students in England. It also broadens evidence from Akbaşlı and Albanna’s (2019) study of academic challenges facing Palestinian students in Turkey.

These findings extend their significance to the broader area of global higher education. They add a new group’s voice to the growing recognition of scholarships as means of individual and potentially national advancement, commonly through service in higher education, e.g., in Ghana, Georgia, and Mongolia (Campbell, 2017; Campbell & Baxter, 2019; Campbell et al., 2021). They outline specific ways through which scholarships yield their significant academic impact, e.g., advanced interdisciplinary specializations and resource-intensive pedagogies which centralize research- and writing-mediated independent and global learning, beyond what is currently (possible to be) at offer in local higher education. The finds further support international evidence of scholarships’ academic impact on recipients’ subject knowledge, learning skills, and overall academic and intellectual capacities for service, inter alia, in higher education (e.g., see Campbell et al., 2021; Demir et al., 2000; Haupt et al., 2021; Jonbekova, 2023; Pikos-Sallie, 2018). Also, they lend support from a new context to the claim that scholarships can represent an inclusive, strategic, and successful investment in the capacity-building of rising academics in/from Global South contexts (Campbell & Basi, 2022; Clift et al., 2013; Dassin et al., 2014). This critical capacity-building potential of scholarships is as significant for higher education recovery and development in Palestine as in other countries in Central and Southeast Asia, Eastern Europe, and West Africa (Campbell, 2017; Campbell et al., 2021; Enkhtur, 2020; Morlang & Stolte, 2008; Singh et al., 2021). In highlighting this potential, the findings finally contribute to illuminating an asset-oriented alternative to the deficit-oriented figure of Global Southern international students (see Madge et al., 2015; Wick et al., 2019). They demonstrate that these international students were not only successful but also appreciative of their learning abroad, though coming from an impoverished higher education background and indeed at first wrestling with their new academic lives. In this case, the “foreign student” is neither failing nor only challenged, but instead is acting on a reflexive agenda of individual advancement and national capacity-building.

## Recommendations

Based on the preceding discussion of findings, I now conclude with a number of recommended actions that may strengthen the potential contribution of scholarships to higher education recovery and development (HERD) in Palestine. I do not address any of these recommendations to a particular group of the many and diverse stakeholders offering, administering, supporting, and/or benefiting from scholarships for Palestinians. I do not do so because more research on Palestinians’ scholarship and post-completion experiences is needed before a determination can be made as to who should do (more of) what. Until then, I find it sufficient to suggest that any stakeholder in a position to act on any of the following recommendations may do so:

1. Scholarships should continue to be offered to Palestinian students, as to others from conflict-affected areas, with the goal of investing in local higher education recovery and development.
2. This offer should not be withheld when the technical value of scholarships does not materialize in the form of immediate contributions to HERD. Scholarships have a strategic value (building capacity for the future) and a moral value (supporting the personal-national aspiration for learning progress despite repeated destruction of learning spaces). Both of these values should be kept in consideration when planning and evaluating scholarship impact.
3. Scholarships to fund Palestinians’ remote study at universities abroad should be welcome. The fact that 40% of the research participants studied in full or in part during COVID-19 lockdowns and still made the academic gains reported in the article may encourage support for this recommendation, which can simultaneously limit risks of brain drain and class- and gender-stratified access to in-person study abroad.
4. The investment through scholarships should continue being inclusive of men and women of different socioeconomic and academic backgrounds, with it in mind that women faculty members at Palestinian and especially Gazan universities continue to be severely underrepresented (Isaac et al., 2019).
5. Pre-departure academic orientation should be offered to scholarship awardees. The goal of this orientation should, at minimum, be to advise them on academic differences and challenges they may encounter in their study destinations. Ideally, this orientation should mark the start of a series of meetings to prompt scholarship recipients to reflect on how they may use their new academic experiences to serve the cause of local higher education recovery and development upon completing their studies abroad.
6. Platforms should be established to bring scholarship alumni together to exchange their experiences, connect with higher education stakeholders, and explore avenues of (coordinated, collaborative) action to support local higher education recovery and development. Virtual settings of such platforms should be welcome as they may mitigate the physical connection barriers caused by the separation between Gaza and the West Bank and by the extension sometimes of scholarship recipients’ sojourn, whether for doctoral study or (academic) work.

Equally importantly, more research should be pursued to better understand the academic and broader impact of scholarships in Palestine, including the affordances and constraints by which it may be shaped. This research should be followed by reflection, including in the platforms mentioned above, with the goal of informing national and international efforts aimed at optimizing the impact of scholarships on local higher education recovery and development.

## Data Availability

The data leading to the research findings presented in this article are not publicly available due to privacy and ethical restrictions. Further inquiries to the corresponding author are welcome.

## Footnotes

1 https://qaddumi.org/en/hani-qaddumi-fund/#baha-1

2 https://saidfoundation.org/scholarships/

3 https://www.amideast.org/our-work/find-a-scholarship/undergraduate-study/hope-fund

4 https://www.alnayzak.org/page.php?id=48351y295761Y48351

5 https://durhampalestine.webspace.durham.ac.uk/scholarships/

6 https://stepspalestine.org.uk/

7 https://www.brookes.ac.uk/alumni-and-supporters/make-a-gift/gaza-scholarship

8 https://www.britishcouncil.ps/en/study-uk/scholarships/hespal
